# Review of Clinical Effects and Presumed Mechanism of Action of the French Oak Wood Extract Robuvit

**DOI:** 10.1089/jmf.2020.0165

**Published:** 2021-09-15

**Authors:** Franziska Weichmann, Fabrice Avaltroni, Carolina Burki

**Affiliations:** Horphag Research, Geneva, Switzerland.

**Keywords:** antioxidant, energy, fatigue, polyphenol, recovery, roburin, supplement, urolithin

## Abstract

Since ancient times, oak wood polyphenols are consumed concomitantly with beverages that are stored and aged in oak wood barrels. Among these polyphenols are roburins, which belong to the class of ellagitannins and only occur in oak. To date, water-extracted standardized French *Quercus robur* wood extract, commercially known as Robuvit^®^, has been investigated in 1172 subjects in over 20 published clinical trials. The results of the clinical studies are consistent with reported effects of urolithins regarding increased mitophagy, pointing to enhanced energy capacity. The Robuvit metabolite urolithin A, B, and C levels and the number of urolithin producers were found to be increased after intake of the extract. Mitophagy is a process, which assigns energy inefficient mitochondria to disassembly, followed by reconstruction to new and more efficient replacements. This effect of Robuvit was observed in different study groups. Supplementation of Robuvit is ascribed to aid chronically fatigued or burnt-out individuals to regain higher energy and activity levels. Robuvit has been further shown to improve conditions such as renal insufficiency, liver insufficiency, mild heart failure, posttraumatic stress disorder and fatigue after surgery and facilitate recovery from mild health impairments such as flu or hangover. There are also indications that Robuvit helps improve erectile function and general loss of vigor in elderly men. *Ex vivo* gene expression experiments using metabolites collected from Robuvit consumers point to increased ribosomal biogenesis in endothelial, neuronal, and keratinocyte cells. Higher ribosome density accelerates the peptide production to meet protein demand, making Robuvit a potential enhancer of physical endurance and performance. A study with recreational athletes, supplemented with Robuvit daily, reported significantly increased performance in triathlon.

## Introduction

Not many plants can provide mankind with food and shelter. Big enough to construct houses, strong enough to build boats resisting ocean travels, and energy-packed to warm up cold winter nights since centuries, the oak tree has historically intermingled with several fundamental aspects of human life. Since antiquity, the oak tree has also granted a more discrete, but direct contribution to human diet. Archetypal European beverages such as wine, sherry, spirits, brandy, whisky, or condiments such as vinegar share oak as the favorite wood type for the barrels, they are slowly matured in a traditional process, which can last up to several decades. During aging, various soluble oak components diffuse into the liquid and, among other properties, enhance the intensity and complexity of its flavors.^1–3^ Another such property is the longer storage life resulting for wines and spirits. This is presumably the primary reason for the practice of aging beverages in oak wood containers. The use for food, such as miso, soy sauce, kimchi, and sauerkraut, attests a protection from spoilage conferred, in part, by the extractables obtained from oak wood containers. Among the different oak species, one is especially praised among winemakers as yielding the highest quality wines: French oak (*Quercus robur* L.).^4^ Conveying benefits of this esteemed oak to humans led to the introduction of Robuvit^®^ standardized French oak wood extract to the market in 2014, manufactured by Horphag Research. To structure and summarize the numerous health benefits of this dietary supplement, evidenced by clinical trials,^5–28^ is the main topic of this review. Its composition, bioavailability, and safety aspects will also be discussed, along with plausible mechanisms of action.

## Composition

**FIG. 1.**
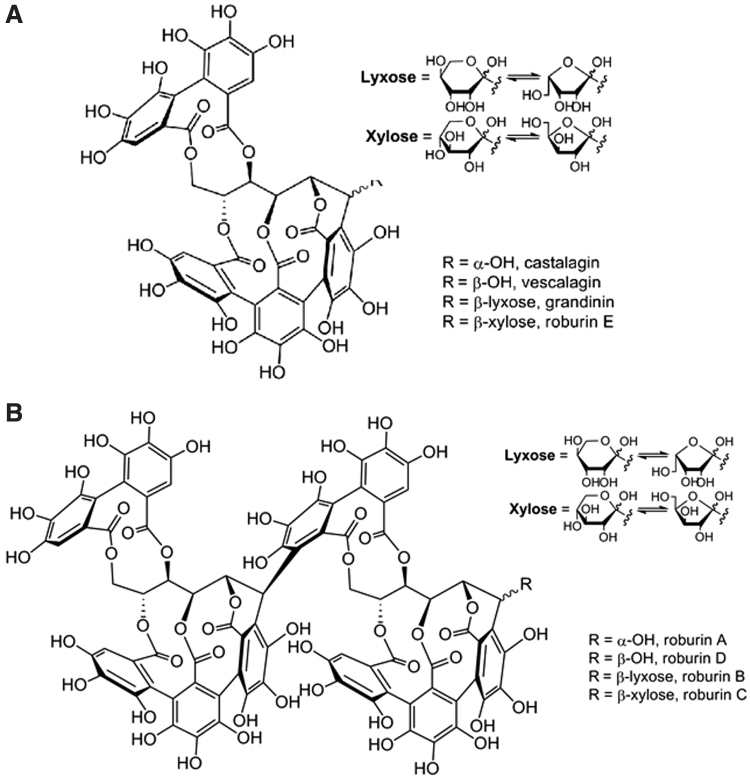
Chemical structures of polyphenolic compounds in Robuvit^®^. **(A)** Chemical structures of castalagin, vescalagin, grandinin, and roburin E. **(B)** Chemical structures of roburins A to D. Reprinted with permission from Natella *et al.*^9^ Copyright (2014) American Chemical Society.

The botanical species used to manufacture Robuvit is *Q. robur* L. (Linnaeus). It belongs to the *Fagacea* family, genus *Quercus*, and is collected in the Massif Central region in France from forests controlled by French agricultural and forestry authorities. This ensures sustainability and respect of stringent environmental protection guidelines. Once gathered, the wood plant part (*lignum*) is extracted using water as the sole solvent. The resulting organoleptic features of Robuvit are a soft characteristic odor and a taste in the woody family notes, more specifically humic, tannic, underwood, and a light beige fine powder appearance. Robuvit is standardized to contain no less than 40% polyphenols, as measured by the well-established Folin–Ciocalteau total phenolic assay.^29^ Its polyphenols belong to the ellagitannin class, named after the ellagic acid moiety esterifying every individual of this class. It possesses potent antioxidant and free-radical scavenging properties.^30^ The ellagitannins determined in Robuvit are the diastereoisomers castalagin and vescalagin (*α*-hydroxyl aglycone and *β*-hydroxyl aglycone, respectively), along with grandinin and roburin E. These compounds differ in their glycosylation patterns (*β*-xylose and *β*-lyxose) (Fig. 1A). The dimeric versions of these structures and signature bioflavonoids specific for *Q. robur* are roburins A, B, C, and D, also differing in their glycosylation patterns (*α*-hydroxyl aglycone, *β*-lyxose, *β*-xylose, and *β*-hydroxyl aglycone) (Fig. 1B).^9^ Other compounds such as castalin, vescalin, and smaller phenolic acids (ellagic acid and gallic acid) are also found in Robuvit.^9^

## Metabolism and Bioavailability

**FIG. 2.**
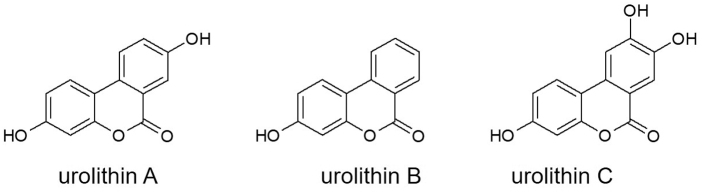
Chemical structures of urolithins in Robuvit. Structure of urolithins A, B, and C, presenting in blood and urine of humans after oral intake of Robuvit.

After initial *in vitro* reports suggested antiatherogenic, antithrombotic, anti-inflammatory, and antiangiogenic effects of ellagitannins, the *in vivo* metabolites of ellagitannins were established to be beneficial in the context of cardiovascular health.^31^ The metabolism of dietary ellagitannins is now known to proceed from ellagic acid through decomposition by gut bacteria into the bioactive metabolites urolithin A, B, and C.^32^ At least one bacterial species present in the human gut microflora specifically involved in this reaction was identified.^33^ Urolithins A, B, and C refer to the 3-mono-, 3,8-di-, and 3,8,9-tri-hydroxyurolithins, respectively, depicted in Figure 2.

**FIG. 3.**
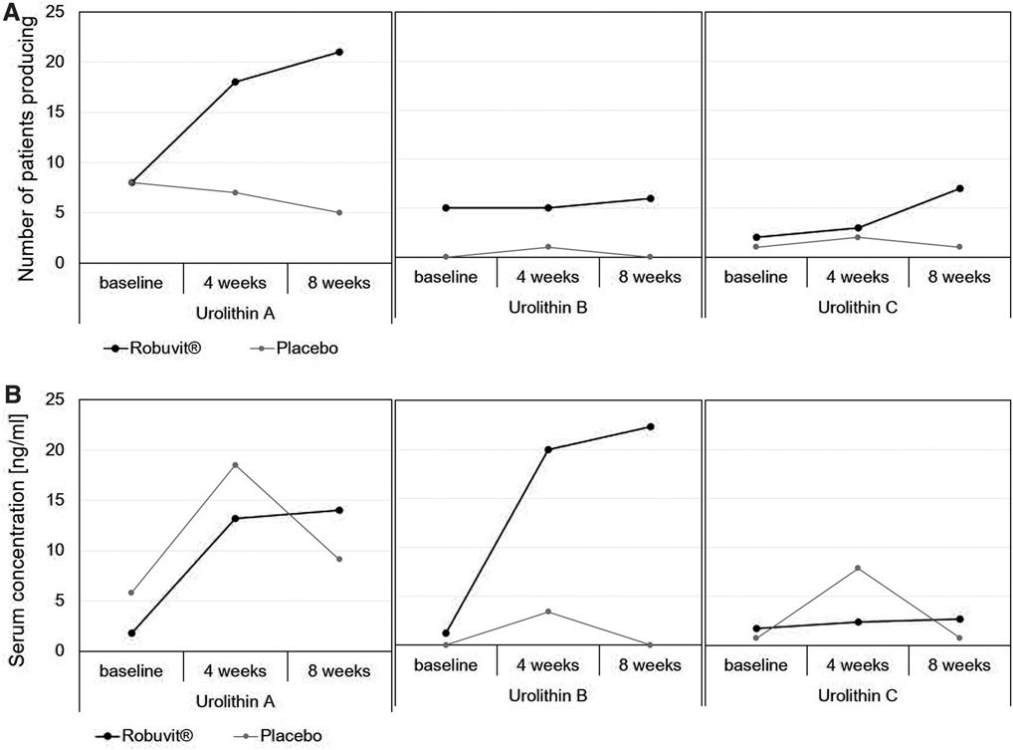
Number of urolithin A, B, and C producers and serum concentrations over time with Robuvit and placebo supplementation. In *black*, the concentrations of the patients taking Robuvit are depicted. In *gray*, the patient's data, supplementing with placebo, are shown. **(A)** The number of patients who produced urolithin A (*left*), B (*middle*), and C (*right*) are given at baseline, after 4 and 8 weeks. The total number of patients included in this study was 66. **(B)** The *left* diagram shows the concentrations of urolithin A in the blood serum of patients at baseline, after 4 and 8 weeks. In the *middle*, urolithin B concentrations are given and the *right* provides the urolithin C amounts in the blood serum. Data from Volpp *et al.*^5^

Robuvit administered three times daily (3 × 100 mg) over a period of 5 days to healthy volunteers was found to significantly increase plasma polyphenols by twofold, coinciding with significant increase in plasma antioxidant capacity.^9^ The volunteers abstained from flavonoid consumption during this time, especially chocolate, fruits, tea, and wine, as much as feasible.^9^ Daily ingestion of Robuvit for 5 days led to the identification of oak roburin metabolites urolithins A, B, and C as well as ellagic acid as glucuronides or diglucuronides.^9^ Furthermore, bioactivity was also demonstrated through an increase of the antioxidant capacity and specific changes of gene expression profile, affecting ribosome, cell cycle, and spliceosome pathways.^9^ In a randomized, double-blind, controlled clinical study, the effect of Robuvit supplementation on patients undergoing hysterectomy was investigated.^5^ Interestingly, the urolithin levels in patients consistently increased over 8 weeks of Robuvit supplementation, which suggests that a chronic intake of Robuvit can induce or amplify the gut bacteria responsible for the urolithin production, leading to an increase in the number of urolithin producers (Fig. 3).^5^

## Safety

Humans have been exposed to oak wood extracts for as long as they have been storing alcoholic beverages and food in oak barrels—that is several millennia in Europe. The safety of Robuvit oak wood extract was tested using the broad array of toxicology studies.^34^ All tests performed concluded that Robuvit is neither genotoxic nor mutagenic. Dermal toxicity tests also revealed that Robuvit is neither irritant nor corrosive and is not a sensitizer. Regarding oral intake, acute and chronic toxicity concluded that a dosage of >700 mg/day is safe, considering European Food Safety Authority guidelines.^35^ Based on this excellent safety profile, Robuvit has also been granted self-affirmed Generally Recognized as Safe status for the United States in 2017 for use in conventional food, by an independent panel of toxicology experts. Published clinical studies used various dosages (up to 600 mg/day) and durations (up to 6 months), depending on the application.^5–28^ To date, a total of 549 patients took Robuvit in medically supervised studies, and no adverse effects have been reported during any of the studies carried out.

## Mechanisms of Action

**FIG. 4.**
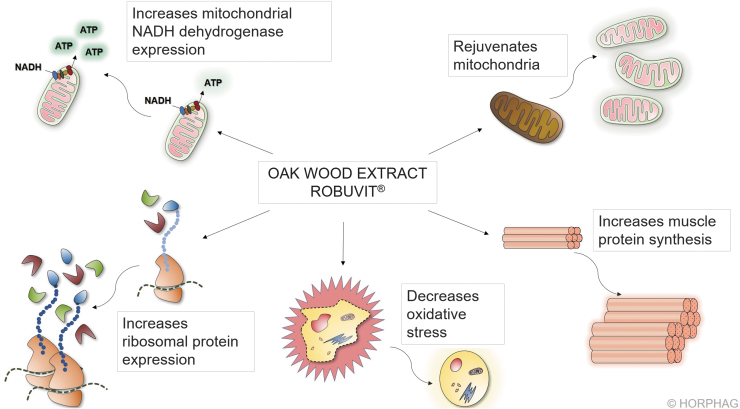
Mechanisms of action of Robuvit. Robuvit has the potential of increasing mitochondrial renewal (mitophagy) via the metabolite urolithin A. Urolithin B, another of Robuvit's metabolites, has been shown to be a regulator of skeletal muscle mass, enhancing growth and differentiation of myotubes. In addition, Robuvit has the potential of decreasing oxidative stress. It was also shown to increase ribosomal protein expression as well as mitochondrial gene expression.

Robuvit affects cellular mitochondria and ribosomes, improving energy and (muscle) protein synthesis and in addition, it decreases oxidative stress (Fig. 4).^9,13,36,37^

### Mitochondria regeneration

Ingested Robuvit polyphenols undergo metabolism by gut microbiota, which generates urolithins A, B, and C (Fig. 1).^9^ The Robuvit metabolite urolithin A favorably affects mitochondria for enhanced energy production in a process referred to as mitophagy.^36,38^ Dysfunctional mitochondria are removed through autophagy, and the turnover of mitochondria is promoted, thus preserving energy metabolism. The mitophagy process ensures that optimal energy in form of adenosine triphosphate (ATP) is gained from consumed food.^36,38^ Mitochondria generating ATP might be also subjected to oxidative damage, which progressively affects the ATP energy output. Mitochondrial regeneration, or mitophagy, is accelerated in presence of Robuvit supplementation and in consequence, a higher energy output may be deployed (Fig. 4, upper right part).^36,38^ In addition to that, a gene expression analysis showed a significant increase in mitochondrial protein nicotinamide adenine dinucleotide (NADH)-dehydrogenase after Robuvit supplementation. This enzyme is directly involved in the electron transport of the respiratory chain to generate ATP and thus improves energy production (Fig. 4, upper left part).^9^

### Increase in muscular mass

Another metabolite found in blood after Robuvit intake—urolithin B—was shown to be a regulator of skeletal muscle mass by inducing muscle hypertrophy (muscle growth), increasing protein synthesis, and enhancing metabolism and energy expenditure (Fig. 4, lower right part).^37^ Stimulated protein synthesis through urolithin B is achieved by activation of the mTORC1 signaling pathway through interaction with the androgen receptor in a nonhormonal way.^37^ This was shown in experiments, conducted in myotubes (multinucleated muscle cells) as well as in *in vivo* experiments.^37^ Urolithin B further leads to the inhibition of the degradation of proteins by downregulation of the ubiquitin proteasome pathway.^37^ In both of these mechanistic effects, urolithin B seems to mimic the effect of testosterone, which acts in a similar way, enhancing muscular mass.^37^

### Ribosomal biogenesis

Clinical research revealed that supplementation with Robuvit in healthy volunteers stimulates ribosomal biogenesis in response to ellagitannin metabolites (Fig. 4, lower left part).^9^ Volunteers' serum samples were investigated *ex vivo* for gene expression modulations in three selected target tissues: endothelium, neuronal, and keratinocyte cell lines.^9^ The results, showing increased expression of ribosomal genes, give rise to the expectation of expanded and accelerated protein synthesis capacity since ribosomes synthesize new proteins from mRNA templates. To date, 24 clinical trials unequivocally point to a significant increase in energy in individuals supplemented with Robuvit, in absence of stimulant effects, as neither heart rate nor blood pressure were reported to be altered.^5–28^ This increased energy generation efficiency is proposed to be the driving force for an enhanced endurance, performance, and faster recovery.

### Increase of antioxidant capacity of blood plasma

A clinical study with 20 healthy volunteers, aged 45–65 years indicated an antioxidant potential of Robuvit (Fig. 4, lower middle part).^13^ Supplementation with 300 mg Robuvit daily for 1 month was ascribed to significantly decrease serum levels of advanced protein oxidation products and lipid peroxides. Interestingly, the study revealed that supplementation with Robuvit significantly stimulated the presence of plasma antioxidant enzymes, such as superoxide dismutase and catalase.^13^ The decreased levels of oxidized proteins and lipid peroxides were found to remain significantly lowered 2 weeks past cessation of Robuvit intake.^13^ A double-blind, placebo-controlled study in 48 patients recovering from surgery confirmed these results.^5^ The patients received 300 mg Robuvit per day or placebo. The levels of oxidized proteins and lipid peroxides were significantly lowered after 4 and 8 weeks compared with placebo and baseline.^5^ In addition, in a study with 40 men aged 50–65 years, the oxidative stress in terms of plasma free radicals, measured in Carr units decreased by 15% compared to the control subjects upon Robuvit supplementation.^24^ Ribosome biogenesis can be affected by hypoxia and oxidative stress as well. Alterations of those mechanisms in ribosome biogenesis are implied in cardiovascular, neurodegenerative, and skeletal disorders.^39^ Since urolithins generated from Robuvit stimulate ribosome biogenesis,^9^ in addition to their antioxidative effects,^5,13,24^ Robuvit may enable an organism to better and more rapidly adjust to challenging situations.

Clinical studies have illustrated the above-mentioned mechanisms of action for the compounds and metabolites of Robuvit in different human populations and health indications. Since most of these studies were pilot studies with a limited number of participants, bigger studies with broader populations are warranted to confirm the effects of Robuvit in an array of specific health markers. Further systematic research on these oak-derived compounds is necessary to uncover the full potential of this new extract.

## Clinical Effects of Robuvit

**FIG. 5.**
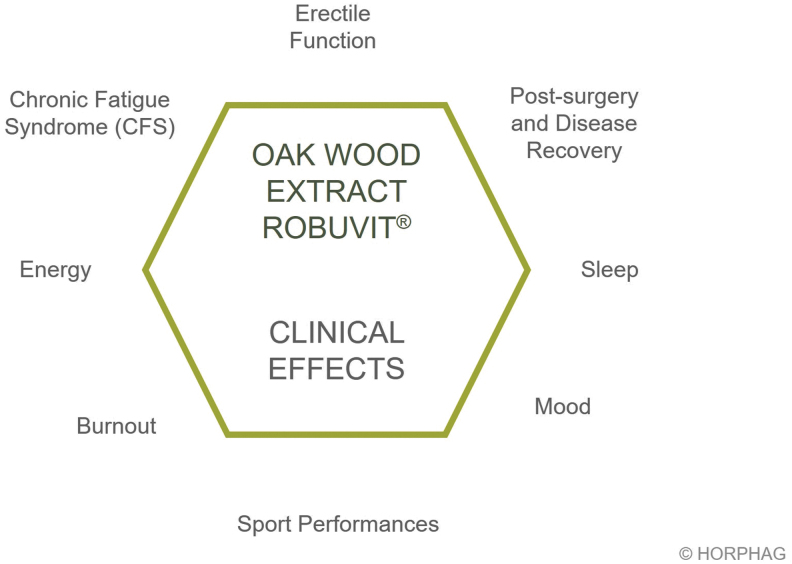
Clinical effects of Robuvit. In several clinical studies, Robuvit supplementation was shown to induce better sleep,^16,23,27^ improve sport performance,^6^ lower symptoms of chronic fatigue syndrome,^22,23^ enhance burnout symptoms,^18^ improve mood^8,16,27^ and erectile function,^22,24^ enhance postsurgery^5,14^ and disease recovery,^7,10–12,17,19,26^ and increase overall energy.^8,22–24^

The reported improved mitochondria regeneration, enhanced ribosomal biogenesis, increased muscle mass, and the elevated antioxidant capacity caused by urolithins give a rational basis for the results of clinical trials with Robuvit, which was shown to accelerate overall energy.^8,22–24^ Decreased fatigue symptoms^22,23^ and better sleep,^16,23,27^ faster recovery after disease^7,10–12,17,19,26^ or surgery,^5,14^ improved mood,^8,16,27^ enhanced erectile function,^22,24^ reinforced antioxidant defense mechanisms in periods of stress,^18^ or enhanced muscle mass gain after intense exercise^6^ are the effects that have been described in diverse clinical studies (Fig. 5). In addition, an ameliorating effect on minimal lymphedema could be observed after supplementation with Robuvit in several studies.^20,21,28^ However, the mechanism of action for this effect is not yet fully elucidated. The antioxidative and anti-inflammatory action of Robuvit^13,24^ could have beneficial impacts in this context, as oxidative stress, enhanced formation of reactive oxygen species and accelerated lipid peroxidation processes are present in chronic lymphedematous tissue.^40,41^

### Increase of energy and decrease of fatigue

**FIG. 6.**
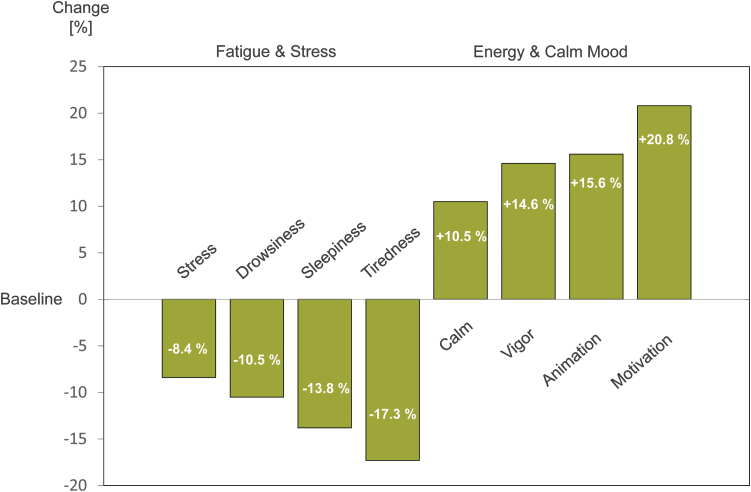
Results of the questionnaire on fatigue and mood. After a 4-week supplementation of Robuvit, 20 healthy individuals from 45 to 65 stated a reduction of stress, drowsiness, sleepiness, and tiredness between 8.4% and 17.3%. Conversely, the survey revealed an increase of calmness, vigor, animation, and motivation between 10.5% and 20.8%. Data from Országhová *et al.*^8^

The earliest study attributing energizing properties to Robuvit supplementation was carried out with 20 healthy individuals aged 45–65 years, neither requiring nor taking medications.^8^ Fatigue and energy were semiquantitatively assessed with the “activation-deactivation, 12-item rating scale,” initially introduced by Thayer.^42^ Study participants refrained from flavonoid consumption, other than Robuvit, during the entire trial period. The applied questionnaire provided participant's energy level subdivided into four categories: “energy,” “calmness,” “tiredness,” and “tension.” Four weeks supplementation with daily 300 mg Robuvit^®^ significantly improved the energy of subjects (*P* < .02), and conversely reduced the tiredness score of study participants (*P* < .02) (Fig. 6).^8^

#### Robuvit improves mood, fatigue, and insomnia

In a clinical study, 40 study participants, who presented with signs and symptoms of fatigue, insomnia, and/or mood alterations, were recruited for a study to identify improvements of symptoms over an observational period of 8 weeks.^16^ Mood tests resulted in a significant improvement in 13 out of 16 mood parameters, rated by the Brief Mood Introspection Scale.^43^ With Robuvit supplementation (300 mg daily), oxidative stress levels decreased significantly after 4 and 8 weeks, fatigue and insomnia scores were also significantly decreased compared to controls. The supplemented subjects slept 33% more and felt much fitter in the morning, being in better mood and with less fatigue during the day.^16^

#### Robuvit increases general vigor

A recent registry study included 40 men, 50–65 years old with self-reported decreased general vigor.^24^ The effect of 300 mg Robuvit per day on the energy status of these men was investigated. After 4 weeks, the antonyms for vigor characteristics, such as apathy, clumsiness, powerlessness, weakness, fatigue, or initial impotence, were all significantly decreased. Properties related to vigor, such as physical and mental health, energy, force, or mental vitality, showed significant improvement. In conclusion, Robuvit supplementation was shown to improve several characteristics of dys-vigor in this study.^24^

#### Robuvit supports energy restoration in chronically fatigued individuals

Robuvit was taken by 48 individuals presenting with signs of exhaustion in chronic fatigue syndrome.^23^ Improvement of symptoms was assessed using a visual analog scale. The results were compared to a group of 43 comparable subjects who did not receive a supplement. The study outcome suggests significant fatigue reduction and a more refreshing sleep after 3 months and further energy restoration after a 6-month daily supplementation with 300 mg Robuvit.^23^ This study further points to improved memory and better ability to concentrate. In another investigation, 38 chronically fatigued individuals were found to respond favorably to 4 weeks supplementation with 300 mg Robuvit daily compared to a matching control group of 42 participants.^22^ The 80 patients were suffering from fatigue since half a year and were at a stage during which taking a rest would not help to overcome exhaustion. Recruited subjects were medically diagnosed for absence of organic and psychologic disorders. Inclusion criteria utilized a questionnaire for chronic fatigue syndrome, which was further used to probe Robuvit supplementation treatment effects after 4 weeks. Robuvit intake was ascribed to significantly alleviate signs and symptoms of fatigue, compared to a control, which did not experience significant improvement of fatigue. Robuvit increased the energy level by 48%.^22^

### Enhanced recovery ability

#### Robuvit metabolites reduce fatigue and promote postsurgical recovery

**FIG. 7.**
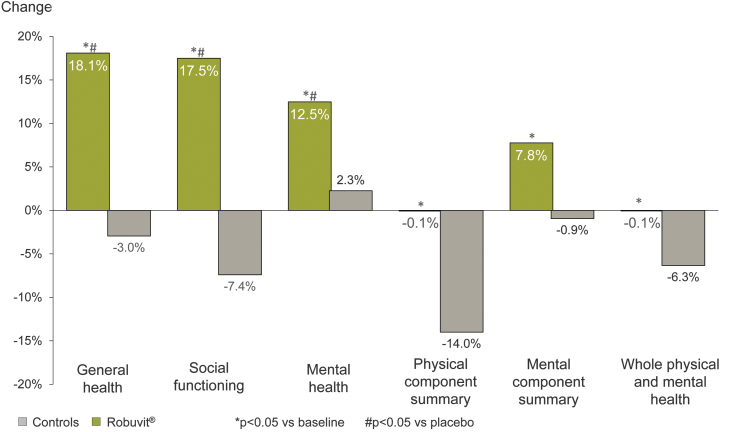
Results of a health survey questionnaire postsurgery. After a hysterectomy, 66 women were either supplemented with Robuvit (300 mg/day) or placebo. After 4 weeks, a health status was given, including general health, social functioning, mental health (all statistically significant compared to baseline [as marked with *] and placebo [as marked with #]), physical component summary, mental component summary, and whole physical and mental health [all statistically significant compared to baseline (as marked with *)]. Data from Ferianec *et al.*^14^

A randomized, double-blind, placebo-controlled, clinical study investigated 66 female subjects who underwent hysterectomy.^5,14^ The group of participating women were randomly assigned to be either supplemented with 300 mg Robuvit or a placebo every day for 8 weeks. The concentration of urolithin metabolites A, B, and C in the serum and blood cells was higher in the supplement group than in the placebo group, who presented with marginal metabolites.^5^ Interestingly, the number of urolithin producers increased over the time of Robuvit intake, which suggests that the gut bacteria, responsible for urolithin production can be induced by regular Robuvit supplementation (Fig. 3A).^5^ A statistically significant association of lower postsurgical pain scores and urolithin A-producing subjects (metabotype A)^44^ was detected (*P* < .05) in the Robuvit group. This shows a direct link between urolithin A and pain relief. A possible explanation for this observation is provided by the anti-inflammatory effects of urolithins.^44^ Cell culture experiments for example showed a downregulating effect of urolithin A on proinflammatory factors, such as nuclear factor kappa-light-chain-enhancer of activated B cells (NF-*κ*B) and cyclooxygenase-2.^45^ The statistical significant association of the urolithin metabotype A and a postoperative recovery score suggests health advantages for those patients, who are urolithin producers in response to Robuvit consumption.^5^ The subjects taking Robuvit showed an improvement in the health survey questionnaire, including general health, social functioning, and mental health after 4 weeks already (Fig. 7).^14^ General health improved by 18% compared to a deterioration of 3% in the placebo group. Social functioning improved by 17.5% with Robuvit and declined by 7.4% in the placebo group and mental health improved by 12.5% with Robuvit and only by 2.3% with placebo. Improvements were statistically significant in the supplement group compared to the placebo group.^14^

#### Supplementation with Robuvit relieves fatigue and accelerates recovery from temporary hepatic dysfunction

In an early registry study, the possible virtues of Robuvit for liver health contributions were investigated in individuals presenting with moderate functional alcoholic hepatic failure, characterized by decreased albumin plasma levels.^7^ Study participants were divided into two comparable groups, 1 with 23 participants, being supplemented with 300 mg Robuvit per day and a second group with 21 participants as control group. Study participants in both groups were investigated over a period of 12 weeks. The results revealed that the group supplemented with Robuvit had significantly lower values of total bilirubin and aspartate transaminase (AST) than the control group, after 6 and 12 weeks.^7^ Daily intake of Robuvit led to a faster and significantly higher increase of albumin levels than in the control group. After 12 weeks, fatigue almost disappeared in the Robuvit group with only 17% of the subjects suffering from fatigue, while it was still present in 43% of the subjects in the control group. The authors concluded that supplementation with Robuvit is valuable for liver health and functionality.^7^ Another investigation with 61 participants presenting with temporary moderate hepatic injury, related to acetaminophen-, antibiotics- or excessive alcohol ingestion exemplifies regenerative properties of Robuvit.^19^ Following a supplementation with 200–300 mg (∼3 mg/[kg·day]) Robuvit per day for 4 weeks, the average total bilirubin values decreased significantly.^19^ After 12 weeks of supplementation with Robuvit, a significant improvement and normal values were reached. Liver enzyme markers alanine transaminase, AST, gamma-glutamyl transferase, C-reactive protein, and erythrocyte sedimentation rate, as well as oxidative stress significantly improved compared to the control group.^19^

#### Robuvit improves renal function and fatigue in kidney insufficiency

Supplementation with 300 mg Robuvit per day over a 4-week period was found to improve kidney function in 57 individuals, presenting with temporary kidney dysfunction.^10^ Study participants were recruited and distributed to either the Robuvit supplementation group or the control group. Fatigue, which was present in all patients at baseline, persisted only in 6.6% of the subjects supplemented with Robuvit, whereas it persisted in 46.6% of the control patients. Study participants with microalbuminuria, who took Robuvit, achieved full normalization of albumin levels.^10^ This shows a significant improvement of the renal function with Robuvit.

#### Robuvit decreases mononucleosis-related fatigue

Mononucleosis represents an infectious disease, predominantly caused by the Epstein-Barr virus, which is commonly transferred by kissing. The symptoms of the infection normally encompass fever, sore throat, lymphadenopathy, and general fatigue.^46^ Signs and symptoms such as a fever and sore throat usually lessen within a couple of weeks, but fatigue, enlarged lymph nodes, and a swollen spleen may last for a few weeks or even months. A group of 50 individuals with mononucleosis were assigned to either the group receiving standard management of the infection, or to another group, receiving same treatment, supplemented with Robuvit 300 mg daily.^12^ Both groups were monitored over a period of 4 weeks. The group, additionally supplemented with Robuvit, showed a statistically significantly improved recovery than the group receiving standard treatment for mononucleosis, particularly regarding the decrease of fatigue.^12^

#### Robuvit improves fatigue due to mild heart failure

Heart failure is a chronic condition, in which the heart does not pump blood as it should. Therefore, transportation of oxygen- and nutrient-rich blood to tissues is reduced. Consequently, affected individuals are rapidly exhausted, even when walking just short distances. To help classify heart failure and guide treatment, ejection fraction is used as an important measurement of the pumping capacity of the heart. Ejection fraction means the percentage of the blood that fills the ventricle and is pumped out with each beat. For an investigation of Robuvit's potential benefits on mild heart failure, 40 study participants with mild, stable heart failure were recruited.^26^ The study participants were either assigned to the group continuing their medications as before or another group supplemented with Robuvit 300 mg/day in addition to their medications over an observational time period of 12 weeks. The ejection fraction in study participants who took Robuvit significantly improved by 7.2% in only 8 weeks, while it did not significantly improve in the control group.^26^ This effect could be explained by the ability of Robuvit to stimulate protein production and skeletal muscle growth. Walking distance on a treadmill was significantly extended in the Robuvit-supplemented group compared with the control group. Finally, fatigue, one of the hallmarks of heart failure, was significantly reduced by Robuvit intake. The score of the modified fatigue impact scale was reduced by more than 54% within 8 weeks in the Robuvit group and remained stable in the control group. The study authors of this pilot study concluded that Robuvit may aid individuals who present with mild heart failure.^26^

#### Robuvit improves fatigue in postflu convalescence

Ippolito *et al.* revealed the efficacy of supplementation with Robuvit for improving fatigue in medical convalescence, after the postdisease period (3 days without disease) subsequent to an influenza infection.^11^ Thirty-eight subjects were included and followed-up for 3 weeks. Eighteen subjects received Robuvit 300 mg/day and 20 subjects served as controls. At baseline, both groups had comparable symptom severity. Compared with controls, subjects supplemented with Robuvit showed significant improvement as early as day 10, and, after 3 weeks, for more symptoms such as weakness, recovery after effort, and alterations in attention and sleep patterns. In addition, performance, heart rate, and oxygen saturation were significantly improved with Robuvit compared with the control group.^11^

#### Robuvit is beneficial for individuals suffering from posttraumatic stress disorder by decreasing fatigue

Supplementation with Robuvit is ascribed to benefit individuals, who suffered a posttraumatic stress disorder (PTSD),^17^ a disorder characterized by failure to recover after experiencing or witnessing a terrifying event. The condition may last months or years, with triggers that can bring back memories of the trauma accompanied by intense emotional and physical reactions. Symptoms may include nightmares or flashbacks, avoidance of situations that bring back the trauma, heightened reactivity to stimuli, anxiety, or depressed mood. A clinical trial investigated earthquake sufferers in Italy, who subsequently to the catastrophe experienced a PTSD.^17^ In all included cases, a well-defined initial traumatic event with multiple fractures occurred, requiring surgery and at least 3 weeks of hospitalization. Volunteering catastrophe survivors of both genders, who agreed to participate in a clinical trial, were supplemented with Robuvit 300 mg/day, whereas a parallel control group received same medical attention, yet in absence of Robuvit supplementation. The study outcome showed that 4 weeks supplementation with Robuvit significantly lowered signs and symptoms of PTSD and decreased the subject's suffering from recurrent memories and nightmares.^17^ Robuvit improved sleep, relieved hypervigilance, intense emotional distress, and emotional numbness and greatly reduced fatigue, enabling people to better cope with PTSD.^17^

#### Robuvit supplementation in subjects with burnout syndrome

An investigation of the effect of Robuvit in 108 participants suffering from burnout, who had significant chronic fatigue, was conducted to identify benefits for individuals living under particularly stressful circumstances.^18^ Forty-two surgeons in training and 66 particularly severely challenged professionals were recruited to identify virtues of Robuvit supplementation. Robuvit intake at the dose of 300 mg/day for 4 weeks significantly improved the burnout symptoms in comparison with the control group.^18^ The symptoms assessed by a questionnaire included coping with problems, feeling positive, being satisfied, and feeling happy at work. In addition, study participants who supplemented with Robuvit experienced a high and significant relieve of emotional drainage, fatigue, level of intolerance, and strain from interactions. The author's conclusions mention a better control of stress management due to fatigue relief.^18^

### Greater endurance in sports

#### Robuvit improves triathlon performance

**FIG. 8.**
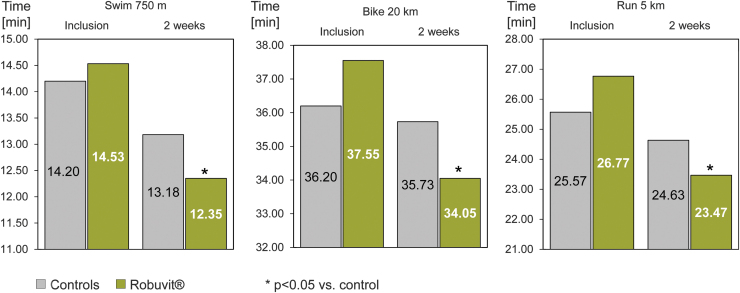
Triathlon performance results following 2 weeks of Robuvit supplementation. The athletes, training for a triathlon performed 750 m swimming, 20 km riding the bike, and 5 km running in the indicated times (in minutes). The *left* of each panel represents the times at inclusion and the *right* shows the times after 2 weeks of training. The *gray bars* show the results of the control group and the *green bars* show the results of the athletes, supplementing with 300 mg Robuvit^®^ per day. *p < 0.05 vs. control. Data from Vinciguerra *et al.*^6^

The contribution of Robuvit on physical expenditure during sport activities was investigated in a controlled trial with 54 recreational sports people of both genders, aged 30–40 years, performing in a triathlon.^6^ One group of 27 participants supplemented with Robuvit 300 mg daily, another 27 comparative individuals were assigned to the control group. Before comparative performances, both groups trained repeatedly together. A significant performance increase after 2 weeks training was identified in subjects with Robuvit supplementation, compared to the corresponding control group, who presented with performance increase as well due to training (Fig. 8).^6^

In addition, this study evaluated the postperformance cramps a day after the triathlon and localized the pain of participants by means of a visual analog rating scale.^6^ Both muscle pain and cramping were described to be significantly lower in the Robuvit supplemented group than in the control group. An interesting observation is that lactate dehydrogenase and unconjugated bilirubin were significantly increased after performance in the control group, while the Robuvit supplemented group presented with no significant alterations of blood hemolysis markers.^6^ These observed effects could be explained by the accelerated mitophagy and the increase in muscle mass. Being facilitated by Robuvit, these mechanisms are suggested to represent the basic contributions for greater physical expenditure in sport activities.

### Amelioration of erectile function

Two different studies suggest a positive effect of Robuvit in sexual function.^22,24^ In a study with 80 patients suffering from chronic fatigue syndrome, 38 of them were supplemented with 300 mg Robuvit per day for 6 months and 42 served as controls.^22^ Already after 4 weeks of supplementation, the participants stated a significant improvement of sexual and relational life by 66%, whereas the control group showed a slight decrease.^22^ Another study on the effects of Robuvit supplementation was conducted, including 40 men between 50 and 65 years who complained of decreased general vigor.^24^ The subjects reported a significant decrease in erectile dysfunction of 38% after 4 weeks, compared to the control men, who stated a decrease of only 10%.^24^ The underlying mechanism of action for this effect was not elucidated yet, but the general increase of energy due to mitophagy, muscle mass increase, and enhanced ribosomal protein expression could explain the ameliorated erectile function. More clinical investigations are needed to confirm the results, but the current data suggest an enhancement of sexual function after Robuvit supplementation.

## Conclusion

Robuvit oak wood extract has been shown to increase overall energy and to reduce fatigue. Ellagitannins, prominent constituents of Robuvit, are metabolized to the bioactive polyphenols urolithins, which are responsible for an elevated mitophagy, the replacement of dysfunctional mitochondria by newer, fully performant ones. It has been shown that the urolithin levels and even the number of urolithin producers increase after Robuvit supplementation. The expression of the mitochondrial enzyme NADH-dehydrogenase was shown to be enhanced after Robuvit supplementation, leading to a more efficient generation of ATP, the principal molecule for storing and transferring energy in cells. Furthermore, Robuvit supplementation leads to improved translation of ribosomal proteins, the cell machinery of crucial importance for producing new proteins. In addition, by stimulating protein synthesis, growth and differentiation of skeletal muscle are facilitated and the muscular mass increases. Finally, Robuvit significantly decreases plasma oxidative stress due to its high antioxidant capacity. Indeed, Robuvit efficacy has been confirmed in a series of clinical studies, including generating energy and reducing fatigue in healthy volunteers, in chronic fatigue syndrome, PTSD, and burn-out patients. It also contributes to improve recovery from flu, mononucleosis, postsurgery, heart failure, and liver and kidney insufficiency. Robuvit improves sport performance, increases general vigor, and ameliorates erectile function. The combination of these versatile effects of French oak wood extract Robuvit is natural prerequisite for greater endurance, recovery, and performance.
